# COVID-19 Pandemic and Fentanyl Use Disorder in African Americans

**DOI:** 10.3389/fnins.2021.707386

**Published:** 2021-08-19

**Authors:** Christopher A. Blackwood, Jean Lud Cadet

**Affiliations:** Molecular Neuropsychiatry Research Branch, NIH/NIDA Intramural Research Program, Baltimore, MD, United States

**Keywords:** COVID-19, fentanyl, FUD, African Americans, opioid use disorders

## Introduction

In the United States (U.S.), the expansion of the use of fentanyl drugs was accompanied by increased numbers of individuals who suffer from fentanyl use disorder (Mori et al., 2006). FUD is a chronic relapsing neuropsychiatric disorder characterized by compulsive drug taking and seeking, despite negative life consequences (DSM-V, 2013). The rise in fentanyl abuse had also led to a surge in fentanyl overdose deaths that were accelerated during the coronavirus disease 2019 (COVID-19) pandemic (CDC, 2020a). These deaths are probably related to the fact that fentanyl and fentanyl analogs have very high affinity for μ-opioid receptors and are therefore extremely toxic when consumed in large quantities because of their interactions with receptors located in brain regions that control respiration. It is important to point out that during the time of increased fentanyl abuse and drug-induced fatal overdoses, there were African American subgroups that have been reported to surpass other ethnic groups in terms of fentanyl-associated overdose deaths (Carlesso and Kara, 2019; Lippold et al., 2019; Chau, 2020; DeLaquil, 2020; Furr-Holden et al., 2021).

People who misuse fentanyl and fentanyl-like drugs often suffer from withdrawal symptoms when access to the drug is not available for an extended period of time (DSM-V, 2013). FUD patients with these symptoms may find it difficult to follow COVID-19-related safety guidelines when in search of opioid drugs to combat withdrawal-associated signs and symptoms. They may also not be able to weigh the benefits of vaccination to prevent the spread of SARS-CoV-2 and SARS-CoV-2 variants throughout their communities. Also of interest is the fact that patients who abuse opioid drugs including fentanyl may be susceptible to abnormalities in organ systems that express opioid receptors including the brain, lungs, and immune systems (Mildh et al., 2001; Borner et al., 2013; Switzer et al., 2020). Consequently, abnormalities in the lungs and immune systems might serve to limit the ability of patients to recover from severe acute respiratory distress syndrome (SARS) observed in some COVID-19 patients (Li and Ma, 2020).

The restrictions introduced during the COVID-19 pandemic have disrupted transit routes along U.S./Mexican border. These disruptions have been reported to impact illegal drug markets including production, trafficking, and consumption of drugs (UNODC, 2020). Public health restriction measures were found to create a scarcity in heroin supplies (UNODC, 2020). Therefore, some drug dealers have pivoted toward mixing street heroin with fentanyl drugs to stretch the supply of heroin to largely unaware opioid users. Since African Americans represent a large percentage of heroin users in the U.S., they are therefore vulnerable to the negative consequences of adulterated heroin supplies.

Herein, we highlight the increasing trend in fentanyl overdose deaths in African American communities and discuss the consequences of fentanyl use on the respiratory system during the COVID-19 pandemic.

## Fentanyl Use Disorder and Medical Vulnerabilities

According to the Centers for Disease Control and Prevention (CDC), there has been a rise in opioid-related overdose deaths, sparked by the use of fentanyl (CDC, 2020a; Mattson et al., 2021). In what appears to be a shift in trend, states and health agencies have reported that subgroups of the African American populations are dying from fentanyl at a higher rate than Caucasians in the U.S. (Carlesso and Kara, 2019; DeLaquil, 2020). Lippold et al. (2019) showed that, in large metropolitan areas, African Americans were found to have a higher rate of overdose deaths involving synthetic opioids, including fentanyl and fentanyl analogs. This study showed that the average number of fentanyl overdose deaths from 2015–2016, compared to that of 2017 showed 56.67% increase in African Americans compared to 46.64% in Caucasians and 52.5% in Latin Americans (Lippold et al., 2019). In the same metro regions, data taken from 2016 to 2017 showed that African Americans between 55 and 64 years of age exhibited the highest (59.53%) fentanyl overdose death rate compared to those found in Caucasians (46.97%) and Latin Americans (17.77%) (Lippold et al., 2019). Another study performed by Spencer and colleagues reported a similar trend when they examined the average rate percentage (ARP) increase of fentanyl overdose deaths from years 2013 through 2015 in comparison to 2016. These observations indicated that all ethnic groups showed increases in ARP although African Americans showed the highest (901.80%) ARP increases of fentanyl overdose deaths in comparisons to Caucasians (382.06%) and Latin Americans (655.03%) (Spencer et al., 2019).

**Figure 1:**
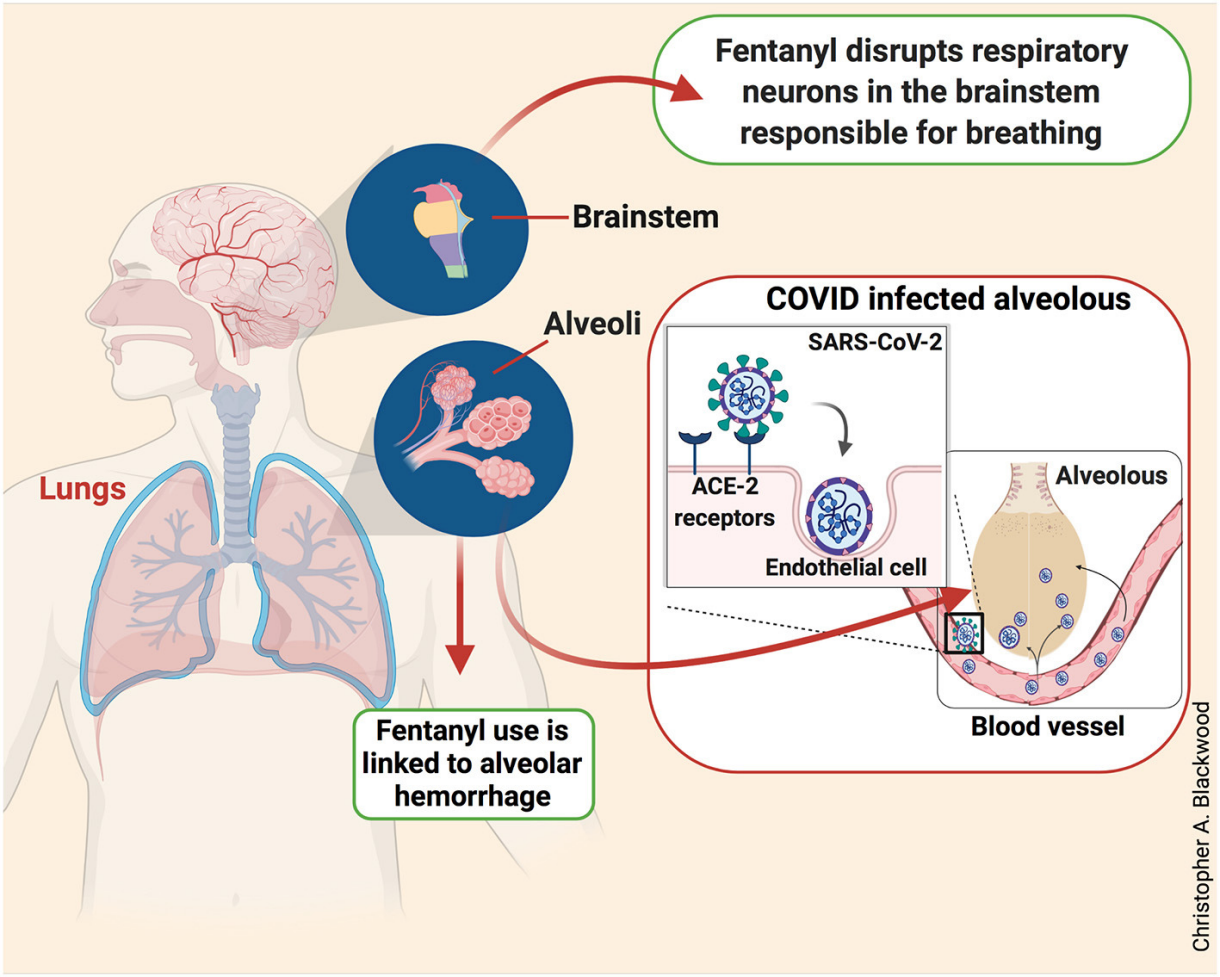
Fentanyl and COVID-19 effects on the respiratory system. Cartoon illustration of a human body showing the negative impacts of fentanyl and SAR-CoV-2 on the respiratory system. Fentanyl disrupts neurons responsible for breathing and causes alveolar hemorrhage in the brainstem and lungs, respectively (green boxes). This illustration also shows how SARS-CoV-2 enters the vascular system through ACE-2 receptors expressed on endothelial cells. The entry of SARS-CoV-2 through endothelial cells is, in part, responsible for the severe acute respiratory syndrome (red box).

African American patients who meet the diagnostic criteria for FUD and also suffer from other medical illnesses could be at high risk of fentanyl-induced death (Hser et al., 2017). Several studies have shown that African Americans have higher rates of lung and cardiovascular diseases compared to other ethnic groups (Torre et al., 2016; Carnethon et al., 2017; Schwartz et al., 2020). Studies performed in animals and humans have reported that intravenous administration of fentanyl negatively affected brainstem respiratory neurons that are responsible for breathing (Tabatabai et al., 1989) as depicted in Figure 1. The disruption of these respiratory neurons has the potential to trigger respiratory depression in humans (Mildh et al., 2001). Furthermore, fentanyl products are associated with several respiratory defects in humans including acute lung injury and diffuse alveolar hemorrhages (Cole et al., 2015; Ruzycki et al., 2016). The causative mechanisms of alveolar hemorrhage may include direct or indirect severe endothelial dysfunctions that are common in opioid toxicity (Dolinak, 2017). Nevertheless, these suggestions need to be further tested.

Patients suffering from SARS have respiratory complications (Li and Ma, 2020) that are related to the ability of SARS-CoV-2 to penetrate endothelial cells through a mechanism that involves angiotensin-converting enzyme-2 (ACE-2) receptors located at the luminal surface of the lungs (Le Bert et al., 2020; Li and Ma, 2020) as shown in Figure 1. Thus, because FUD patients are also vulnerable to severe respiratory complications due to the negative effects of the drug on the brainstem and lungs, those patients need to be closely monitored since the abuse of fentanyl-like products could exacerbate health outcomes in the presence of severe COVID-19 signs and symptoms.

## COVID-19 and African American Communities

According to COVID-19 mortality records from the National Vital Statistics System, African Americans showed disproportionately higher percentage of COVID-19-related death (CDC, 2020b). In Washington D.C., African Americans represented roughly 44% of the population, but account for ~69.2% of all COVID-19-related deaths. Similarly, in the states of Louisiana and Mississippi, African Americans comprised ~33.0 and 31.0% (respectively), of their total population, but represented ~59.0 and ~40.1% of all COVID-19-related deaths (CDC, 2020b; LDOH, 2020; Price-Haywood et al., 2020). Although, fentanyl overdose deaths increased after the first cases of COVID-19 were identifies in the U.S. (CDC, 2020a,c), there has been very little progress to determine the impact of fentanyl use and COVID-19 deaths in the general population and in African American patients, specifically. These trends need to be monitored more closely because of their potential impact on the clinical course of COVID-19 and potential changes in approaches to the vaccination of patients with opioid use disorders since both fentanyl (Borner et al., 2013) and SARS-CoV-2 (Chen and Wherry, 2020) negatively impact the respiratory system.

## Recommendations

The rise in fentanyl overdose deaths in African American communities is alarming and suggests that new strategies tailored to mitigate the problem in this population are needed. We recommend the expansion of awareness programs about the risks of fentanyl in the drug supply, the expansion of naloxone distribution for overdose reversal, and increased access to drug treatment programs. We believe that multi-level teams should include community leaders, policy makers, government agencies, educators, prevention specialists, and treatment and recovery providers are needed to develop tailored intervention strategies targeted toward the treatment barriers that are prevalent in African American communities. Furthermore, following long periods of abstinence, recent inmates and patients being treated for an opioid use disorder might benefit from overdose prevention educational programs and take-home naloxone for themselves and others who might be witnesses to an overdose episode (McDonald and Strang, 2016; Lambdin et al., 2020).

There is a need to address the health inequities in the African American community. The reasons for these disparities have thought to include long-standing systemic health and social inequities, as well as, the structural racism, which if not addressed, may subsequently elevate COVID-19 death rates in other states with large populations of African Americans. We recommend programs tailored to expand COVID-19 vaccination in these marginalized communities to mitigate the risk of dying from COVID-19. These should include the placement of large movable vehicles within communities with poor public transportation.

To conclude, we recommend that more studies be conducted to identify health inequities that exist in African American communities as far as opioid prevention, interventions, and treatment resources are concerned. The under-representation of African Americans in medical research is a major impediment in addressing the various health disparities in fentanyl and COVID-19-related deaths in these communities. Erosion of trust due to past unethical clinical trials/research studies conducted on African Americans (Scharff et al., 2010; Truog et al., 2012; Hoffman et al., 2016; Wailoo, 2018) likely contributed to their low enrollment in research studies. Furthermore, we believe that there is an acute need to address the lack of representation and diversity among research investigators conducting studies in African American communities. Indeed, several studies have reported that diversity in research teams leads to more accurate decisions and problem solving (Antonio et al., 2004; Sommers, 2006; Philips, 2017). Finally, increasing diversity on research teams is indeed an obligatory first step to address the long lasting health inequities in the African American community.

## Author Contributions

CB drafted and conceptualized the manuscript. JC reviewed and edited the contents of the paper. Both authors approved the final draft of the manuscript.

## Conflict of Interest

The authors declare that the research was conducted in the absence of any commercial or financial relationships that could be construed as a potential conflict of interest.

## Publisher's Note

All claims expressed in this article are solely those of the authors and do not necessarily represent those of their affiliated organizations, or those of the publisher, the editors and the reviewers. Any product that may be evaluated in this article, or claim that may be made by its manufacturer, is not guaranteed or endorsed by the publisher.

## References

[B1] Antonio A. L. Chang M. J. Hakuta K. Kenny D. A. Levin S. Milem J. F. (2004). Effects of racial diversity on complex thinking in college students. Psychol. Sci. 15, 507–510. 10.1111/j.0956-7976.2004.00710.x 15270993

[B2] Borner C. Lanciotti S. Koch T. Hollt V. Kraus J. (2013). Mu opioid receptor agonist-selective regulation of interleukin-4 in T lymphocytes. J. Neuroimmunol. 263, 35–42. 10.1016/j.jneuroim.2013.07.012 23965172

[B3] Carlesso K. J. Kara J. (2019). Best of 2019: Blacks Dying Form Fentanyl at Same Rate as Whites for the First Time. Hartford, CT: The CT mirror.

[B4] Carnethon M. R. Pu J. Howard G. Albert M. A. Anderson C. A. M. Bertoni A. G.. (2017). Cardiovascular health in African Americans: A scientific statement from the American Heart Association. Circulation 136, e393–e423. 10.1161/CIR.0000000000000534 29061565

[B5] CDC (2020a). Increase in Fatal Drug Overdoses Across the United States Driven by Synthetic Opioids Before and During the COVID-19 Pandemic. Atlanta, GA: Centers for Disease Control and Prevention (CDC); U.S. Department of Health and Human Services.

[B6] CDC (2020b). Distribution of COVID-19 Deaths and Population Distributions by Race and Ethnicity. Centers for Disease Control and Prevention, National Center for Health Statistics. Atlanta, GA: Centers for Disease Control and Prevention (CDC); U.S. Department of Health and Human Services.

[B7] CDC (2020c). First Travel-Related Case of 2019 Novel Coronavirus Detected in United States. Atlanta, GA: Centers for Disease Control and Prevention (CDC); U.S. Department of Health and Human Services.

[B8] Chau V. (2020). Substance Abuse and Mental Health Services Administration: The Opioid Crisis and the Black/African American Population: An Urgent Issue. Rockville, MD: U.S. Department of Health and Human Services.

[B9] Chen Z. Wherry J. E. (2020). T cell responses in patients with COVID-19. Nat. Rev. Immunol. 20, 529–536. 10.1038/s41577-020-0402-6 32728222 PMC7389156

[B10] Cole J. B. Dunbar J. F. McIntire S. A. Regelmann W. E. Slusher T. M. (2015). Butyrfentanyl overdose resulting in diffuse alveolar hemorrhage. Pediatrics 135, e740–e743. 10.1542/peds.2014-2878 25713275

[B11] DeLaquil M. (2020). Differences in Rates of Drug Overdose Deaths by Race. St. Paul, MN: Minnesota Health Department.

[B12] Dolinak D. (2017). Opioid toxicity. Acad. Forensic Pathol. 7, 19–35. 10.23907/2017.003 31239953 PMC6474471

[B13] DSM-V (2013). Diagnostic and Statistical Manual of Mental Disorders. Washington, DC: American Psychiatric Association. 10.1176/appi.books.9780890425596

[B14] Furr-Holden D. Milam A. J. Wang L. Sadler R. (2021). African Americans now outpace Whites in opioid-involved overdose deaths: a comparison of temporal trends from 1999 to 2018. Addiction 116, 677–683. 10.1111/add.15233 32852864

[B15] Hoffman K. M. Trawalter S. Axt J. R. Oliver M. N. (2016). Racial bias in pain assessment and treatment recommendations, and false beliefs about biological differences between blacks and whites. Proc. Natl. Acad. Sci. U.S.A. 113, 4296–4301. 10.1073/pnas.1516047113 27044069 PMC4843483

[B16] Hser Y. I. Mooney L. J. Saxon A. J. Miotto K. Bell D. S. Zhu Y.. (2017). High mortality among patients with opioid use disorder in a large healthcare system. J. Addict. Med. 11, 315–319. 10.1097/ADM.0000000000000312 28426439 PMC5930020

[B17] Lambdin B. H. Bluthenthal R. N. Wenger L. D. Wheeler E. Garner B. Lakosky P.. (2020). Overdose education and naloxone distribution within syringe service programs - United States, 2019. MMWR Morb. Mortal. Wkly. Rep. 69, 1117–1121. 10.15585/mmwr.mm6933a2 32817603 PMC7439981

[B18] LDOH (2020). Louisiana Department of Health (L.D.O.H) Office of Public Helath. Baton Rouge, LA.

[B19] Le Bert N. Tan A. T. Kunasegaran K. Tham C. Y. L. Hafezi M. Chia A.. (2020). SARS-CoV-2-specific T cell immunity in cases of COVID-19 and SARS, uninfected controls. Nature 584, 457–462. 10.1038/s41586-020-2550-z 32668444

[B20] Li X. Ma X. (2020). Acute respiratory failure in COVID-19: is it “typical” ARDS? Crit. Care 24:198. 10.1186/s13054-020-02911-9 32375845 PMC7202792

[B21] Lippold K. M. Jones C. M. Olsen E. O. Giroir B. P. (2019). Racial/ethnic and age group differences in opioid and synthetic opioid-Involved overdose deaths among adults aged >/=18 years in metropolitan areas - United States, 2015-2017. MMWR Morb. Mortal. Wkly. Rep. 68, 967–973. 10.15585/mmwr.mm6843a3 31671083 PMC6822810

[B22] Mattson C. L. Tanz L. J. Quinn K. Kariisa M. Patel P. Davis N. L. (2021). Trends and geographic patterns in drug and synthetic opioid overdose deaths - United States, 2013-2019. MMWR Morb. Mortal. Wkly. Rep. 70, 202–207. 10.15585/mmwr.mm7006a4 33571180 PMC7877587

[B23] McDonald R. Strang J. (2016). Are take-home naloxone programmes effective? Systematic review utilizing application of the Bradford Hill criteria. Addiction 111, 1177–1187. 10.1111/add.13326 27028542 PMC5071734

[B24] Mildh L. H. Scheinin H. Kirvela O. A. (2001). The concentration-effect relationship of the respiratory depressant effects of alfentanil and fentanyl. Anesth. Analg. 93, 939–946. 10.1097/00000539-200110000-00028 11574361

[B25] Mori H. Ninomiya K. Kino-oka M. Shofuda T. Islam M. O. Yamasaki M.. (2006). Effect of neurosphere size on the growth rate of human neural stem/progenitor cells. J. Neurosci. Res. 84, 1682–1691. 10.1002/jnr.21082 17044035

[B26] Philips K. W. (2017). How Diversity Makes us Smarter. Berkeley, CA: Greater Good Magazine.

[B27] Price-Haywood E. G. Burton J. Fort D. Seoane L. (2020). Hospitalization and mortality among black patients and white patients with COVID-19. N. Engl. J. Med. 382, 2534–2543. 10.1056/NEJMsa2011686 32459916 PMC7269015

[B28] Ruzycki S. Yarema M. Dunham M. Sadrzadeh H. Tremblay A. (2016). Intranasal fentanyl intoxication leading to diffuse alveolar hemorrhage. J. Med. Toxicol. 12, 185–188. 10.1007/s13181-015-0509-5 26503098 PMC4880598

[B29] Scharff D. P. Mathews K. J. Jackson P. Hoffsuemmer J. Martin E. Edwards D. (2010). More than Tuskegee: understanding mistrust about research participation. J. Health Care Poor Underserved 21, 879–897. 10.1353/hpu.0.0323 20693733 PMC4354806

[B30] Schwartz N. G. Price S. F. Pratt R. H. Langer A. J. (2020). Tuberculosis - United States, 2019. MMWR Morb. Mortal. Wkly. Rep. 69, 286–289. 10.15585/mmwr.mm6911a3 32191684 PMC7739979

[B31] Sommers S. R. (2006). On racial diversity and group decision making: identifying multiple effects of racial composition on jury deliberations. J. Pers. Soc. Psychol. 90, 597–612. 10.1037/0022-3514.90.4.597 16649857

[B32] Spencer M. R. Warner M. Bastian B. A. Hedegaard H. (2019). Drug overdose deaths involving fentanyl, 2011-2016. Natl. Vital. Statist. Rep. 68:9. 31112123

[B33] Switzer A. R. Beland B. Sarna J. R. Walzak A. Pfeffer G. (2020). Fentanyl overdose causing hippocampal ischaemia followed by delayed leukoencephalopathy. Can. J. Neurol. Sci. 47, 398–399. 10.1017/cjn.2020.33 32063243

[B34] Tabatabai M. Kitahata L. M. Collins J. G. (1989). Disruption of the rhythmic activity of the medullary inspiratory neurons and phrenic nerve by fentanyl and reversal with nalbuphine. Anesthesiology 70, 489–495. 10.1097/00000542-198903000-00020 2923296

[B35] Torre L. A. Siegel R. L. Jemal A. (2016). Lung cancer statistics. Adv. Exp. Med. Biol. 893, 1–19. 10.1007/978-3-319-24223-1_1 26667336

[B36] Truog R. D. Kesselheim A. S. Joffe S. (2012). Research ethics. Paying patients for their tissue: the legacy of Henrietta Lacks. Science 337, 37–38. 10.1126/science.1216888 22767914 PMC4256075

[B37] UNODC (2020). United Nation Office on Drugs and Crime: COVID-19 and the Drug Supply Chain: From Production and Trafficking to Use. Vienna.

[B38] Wailoo K. (2018). Historical aspects of race and medicine: The case of J. Marion Sims. JAMA 320, 1529–1530. 10.1001/jama.2018.11944 30264160

